# Contemporary Treatment Patterns and Response in Relapsed/Refractory Cutaneous T-Cell Lymphoma (CTCL) across Five European Countries

**DOI:** 10.3390/cancers14010145

**Published:** 2021-12-29

**Authors:** Chalid Assaf, Nathalie Waser, Martine Bagot, Mary He, Tina Li, Mehul Dalal, Francois Gavini, Fabrizio Trinchese, Athanasios Zomas, Meredith Little, Nicola Pimpinelli, Pablo L. Ortiz-Romero, Timothy M. Illidge

**Affiliations:** 1 Department of Dermatology, HELIOS Klinikum Krefeld, Academic Teaching Hospital of the University of Aachen, 47805 Krefeld, Germany; 2 Department of Dermatology, Charité-Universitätsmedizin, 10117 Berlin, Germany; 3 ICON Plc, 450-688 West Hastings St., Vancouver, BC V6B 1P1, Canada; Nathalie.Waser@iconplc.com (N.W.); maryhe0229@me.com (M.H.); tina.ljj@gmail.com (T.L.); 4 Department of Dermatology, Hôpital Saint-Louis, 75010 Paris, France; martine.bagot@aphp.fr; 5 Takeda Development Center Americas, Inc. (TDCA), Lexington, MA 02139, USA; mehul.dalal@takeda.com (M.D.); francois.gavini@takeda.com (F.G.); Meredith.little@gmail.com (M.L.); 6 Takeda Pharmaceuticals International AG, 8152 Zurich, Switzerland; fabrizio.trinchese@gmail.com (F.T.); Athanasios.Zomas@takeda.com (A.Z.); 7 Department of Health Sciences, Dermatology Unit, University of Florence, 50121 Florence, Italy; nicola.pimpinelli@unifi.it; 8 Institute I+12, Medical School, Hospital Universitario 12 de Octubre, University Complutense, 28040 Madrid, Spain; portiz.hdoc@salud.madrid.org; 9 Manchester NIHR Biomedical Research Centre, Christie Hospital, University of Manchester, Manchester M20 4BX, UK; tim.illidge@manchester.ac.uk

**Keywords:** CTCL, pcALCL, treatment, observational study, outcome, TTNT, OS

## Abstract

**Simple Summary:**

Recent studies have mostly focused on the treatment of advanced mycosis fungoides and Sézary syndrome and less on the treatment of relapsed or refractory (R/R) cases of cutaneous T-cell lymphoma (CTCL). There is also a paucity of data on the treatment and outcomes of primary cutaneous anaplastic large cell lymphoma and other rare CTCL subtypes. This European observational study provided real-world data on the current treatments used in the management of CTCL across three lines of therapy. As a result, there was a significant level of heterogeneity in treatment types than expected by European guidelines. These findings highlight the lack of consensus in relapsed CTCL, as well as the urgent unmet treatment needs.

**Abstract:**

The treatment pattern of cutaneous T-cell lymphoma (CTCL) remains diverse and patient-tailored. The objective of this study was to describe the treatment patterns and outcomes in CTCL patients who were refractory or had relapsed (R/R) after a systemic therapy. A retrospective chart review study was conducted at 27 sites in France, Germany, Italy, Spain and the United Kingdom (UK) of patients who received a first course of systemic therapy and relapsed or were refractory. Data were collected longitudinally from diagnosis to first-, second- and third-line therapy. The study included 157 patients, with a median follow-up of 3.2 years. In total, 151 proceeded to second-line and 90 to third-line therapy. In the first line (*n* = 147), patients were treated with diverse therapies, including single- and multi-agent chemotherapy in 67 (46%), retinoids in 39 (27%), interferon in 31 (21%), ECP in 4 (3%), corticosteroids in 3 (2%) and new biological agents in 3 (2%). In the second line, the use of chemotherapy and retinoids remained similar to the first line, while the use of new biologics increased slightly. In sharp contrast to the first line, combination chemotherapy was extremely diverse. In the third line, the use of chemotherapy remained high and diverse as in the second line. From the time of first R/R, the median PFS was 1.2 years and the median OS was 11.5 years. The presented real-world data on the current treatments used in the management of R/R CTCL in Europe demonstrate the significant heterogeneity of systemic therapies and combination therapies, as expected from the European guidelines.

## 1. Introduction

Cutaneous T-cell lymphoma (CTCL) is a rare form of non-Hodgkin lymphoma (NHL) involving T-lymphocytes of the skin, accounting for 4% of all NHLs [1]. CTCL is a debilitating hematological malignancy in the skin lymphoid tissue, for which treatment remains diverse and patient-tailored [1,2,3,4]. Multiple subtypes of CTCL exist; the two most common subtypes are mycosis fungoides (MF) and Sézary syndrome (SS), which together account for 65% of all CTCL cases [1]. Over time, the reported incidence of CTCL has been steadily increasing. In Europe, the incidence of CTCL is approximately 1200 new cases per year, with a prevalence of approximately 16,000 cases [5,6]. The disease is more common among men and has a peak incidence among people between the ages of 70 and 80 years [5,7].

Treatment plans for CTCL are variable and dependent on the extent and anatomical location of skin involvement, type of skin lesions and whether the lymphoma has extracutaneous involvement [8,9,10]. With the exception of an allogeneic stem cell transplant and localized radiotherapy in unilesional MF, there are no curative therapies for this disease [11,12]. Therefore, therapy is multimodal and takes place over the course of a lifetime [12]. Systemic therapy is typically deferred until patients fail to respond to skin-directed therapies [12,13]. Therapy also depends on the subtype of CTCL [10]. Patients with primary cutaneous anaplastic large-cell lymphoma (pcALCL) typically present with localized tumors or nodules that are treated with local radiotherapy (RT) or surgical excision [10]. Patients with early-stage MF typically start with skin-directed therapy and local RT when infiltrated plaques occur [10]. Patients refractory to local skin-directed therapy go on to receive a combination of psoralen and ultraviolet A (PUVA) plus systemic therapy, e.g., interferon (IFN) alpha, PUVA plus retinoids, IFN-alpha plus retinoids or total-skin electron-beam irradiation (TSEB) [10]. For patients with advanced and/or refractory disease, systemic mono chemotherapy with methotrexate, gemcitabine or liposomal doxorubicin can be initiated [10].

Newer agents such as histone deacetylase (HDAC) inhibitors or low-dose folate inhibitors can also be used for systemic therapy in some countries [9]. Patients with SS have systemic disease at onset and are treated with skin-directed therapy (such as PUVA or a potent topical steroid) as adjuvant therapy. Other options considered for patients with systemic SS include extracorporeal photopheresis (ECP), chlorambucil plus prednisone, methotrexate, ECP alone or in combination with IFN-alpha, retinoid, TSEB or PUVA and systemic therapies in combination with PUVA (either IFN-alpha or retinoids) [8,10,14]. Second-line therapy for SS includes alemtuzumab, gemcitabine, pegylated liposomal doxorubicin, mogamulizumab, CHOP (i.e., cyclophosphamide, doxorubicin, vincristine, prednisone), CHOP-like polychemotherapies and other multi-agent chemotherapy. Recently, CD30-targeting agents have been investigated and approved as a therapy for CTCL and may create new treatment opportunities for this disease [15,16,17].

With new, emerging treatments for relapsed/refractory (R/R) CTCL, it is important to understand the current treatment patterns in the real-world setting. A retrospective chart review was conducted to investigate these treatment patterns in CTCL patients who have relapsed or are refractory after at least one prior systemic therapy in France, Germany, Italy, Spain and the United Kingdom (UK). This study contributes real-world data to the knowledge gap regarding patient characteristics, treatment patterns and outcomes (effectiveness, toxicity, resource utilization) in the management of patients with R/R CTCL.

## 2. Materials and Methods

A multicenter, retrospective chart review was conducted from June 2017 to November 2018 in patients diagnosed with CTCL who had relapsed or were refractory following at least one prior systemic therapy (or prior RT for patients with pcALCL). Sites were selected based on a list of centers known to treat CTCL patients in France, Germany, Italy, Spain and the UK. Following a feasibility assessment, 27 sites were selected to contribute a representative sample in terms of type of center (i.e., community vs. academic), specialty of caring physician and geography while, at the same time, recognizing the practicality of recruiting a sufficiently large sample.

Eligible patients were adults 18 years of age and older, with a physician-confirmed CTCL diagnosis, who received a first course of systemic therapy (except pcALCL) and relapsed or were refractory prior to 1 January 2016. For pcALCL, eligible patients were adults 18 years of age and older, with a physician-confirmed diagnosis, who received a first course of systemic therapy or RT and became R/R prior to 1 January 2016. Patients whose systemic therapy or RT prior to being R/R was administered as part of a clinical trial were excluded. Eligible patients were managed according to local practices by dermatologists, hematologists, oncologists and radiologists at 27 sites in France (4), Germany (4), Italy (7), Spain (7) and the UK (5). Sites included academic teaching hospitals (68.8%), cancer centers (14.7%) and community centers (15.9%). Sites screened patients for eligibility using medical charts of living or deceased patients with CTCL. In terms of subtypes, patients with pcALCL were of particular interest and thus preferentially selected for inclusion when available.

Data from the patients’ medical charts were extracted by the sites into an electronic data capture (EDC) system. Data included patient demographics and clinical, histological and genetic characteristics, i.e., CTCL subtype, TNMB stage, CD30 and T-cell receptor (TCR) gene rearrangement. Treatments in 1st, 2nd and 3rd lines were captured. A line of therapy was defined as all treatments received from the time of treatment initiation to the time of R/R as determined by the treating physician. Treatment types of particular focus included local skin-directed therapy, systemic therapy and RT, but presence of hematopoietic stem cell transplantation (HSCT) and treatment administered within a clinical trial in 2nd and 3rd line were also captured succinctly. Data related to local skin-directed therapy were restricted to the type of treatment, i.e., topical therapy, PUVA, UVB/narrow band UVB phototherapy and photodynamic therapy. Data related to systemic therapy included the type of treatment and regimen(s), the start and end dates and any discontinuation and reason for discontinuation. Data related to RT included the type of RT (i.e., electron beam vs. X-ray), the start and end date, the extent of exposure (i.e., local vs. total) and the target location (i.e., arms, head, face, posterior and anterior trunk, legs and palms). Disease stage was obtained from patients’ charts according to and based on TNMB staging. For patients with MF and SS, stages I-IV were determined based on TNMB staging according to the International Society for Cutaneous Lymphomas (ISCL) and European Organization for Research and Treatment of Cancer (EORTC) proposed classification [2,18].

Each patient was assigned a unique identifier in the EDC. Sites had access to the patients’ charts and the key linking the unique identifiers to patients’ charts, but not the sponsor or contract research organization. Inconsistent or illogical entries (including dates) triggered alerts in the EDC. If the illogical entries could not be resolved in the EDC by the site staff, sites were contacted by phone or email using the unique identifiers to query the data. A data management plan pre-specified critical variables (i.e., year of birth, year of diagnosis, primary CTCL subtype at diagnosis, dates of R/R), range checks and the query generation process.

Central or local ethics approvals were obtained prior to performing data collection, in accordance with local regulations. The study was approved by the following central ethics committees and data protection agencies in France and the UK:

- The London Riverside Research Ethics Committee (on 25 May 2017) and Health Research Authority (HRA) (on 26 May 2017);
- Comité Consultatif sur le Traitement de l’Information en matière de Recherche dans le domaine de la Santé (CCTIRS) (on 6 June 2017);
- La Commission Nationale Informatique et Libertés (CNIL) (on 8 December 2017).

The list of local ethics committees in Germany, Italy and Spain is provided in the Institutional Review Board Statement.

The study was descriptive in nature and thus no formal sample size calculations were performed. The aim was to achieve a target of 40 patients per country (200 patients total). Systemic treatments were categorized using a hierarchical classification to allow per-patient summaries when multiple systemic therapies were administered in one line of therapy. In this classification, chemotherapy (including conventional chemotherapy and methotrexate) was considered to be the highest level of category. Retinoids, IFN and corticosteroids were considered to be second, third and fourth levels, respectively. Finally, ECP, HDAC inhibitors, alemtuzumab, bortezomib and brentuximab vedotin were considered categories of their own, regardless of any other therapy administered.

Outcomes included clinical response, skin symptoms, safety/toxicity, time to R/R, overall survival (OS), progression-free survival (PFS) and resource utilization. Adverse events (AEs) included all treatment-related and non-treatment related AEs. Clinical response was assessed by radiological criteria and included complete response, partial response, stable disease and progressive disease. Global response was based on scoring of TNMB, i.e., skin, lymph nodes, viscera and blood, respectively [2]. Skin response alone was recorded when global response was not available in the patient’s chart. Time to R/R was defined as time from systemic therapy initiation or RT initiation to time of R/R. OS was defined as time from first R/R (i.e., index date) to death, last known vital status or loss to follow-up, whichever occurred first. PFS was defined as time from index date to clinical progression, relapse, refractory status, subsequent therapy, death or loss to follow-up, whichever occurred first.

Statistical analysis was conducted in R 3.4.1 and data were summarized using descriptive statistics. Data were presented as means, standard deviations (SDs), medians, interquartile (IQR) range and ranges for continuous variables; numbers and percentages were used for categorical variables. For data, month and year were required, and missing days were imputed as 15. OS and PFS plots were generated using Kaplan–Meier analysis. Median OS and PFS were presented including 95% confidence intervals (CI).

## 3. Results

A total of 157 patients were included in this retrospective study (i.e., 19 in France, 21 in Germany, 40 in Italy, 39 in Spain and 38 in the UK). Among these 157 patients, the majority were diagnosed with CTCL between 2011 and 2016 (53.5% in 2011–2016, 28.7% in 2006–2010, 10.2% in 2001–2005 and 7.6% in 1981–2000). The median follow-up time from diagnosis was 5.6 years (range: 0.6–28.4). Patient demographics and clinical characteristics at diagnosis are summarized in Table 1. At the time of diagnosis, the median age was 56.0 years (range: 19.0–97.0), the male to female ratio was 1.7 and the predominant ethnicity was Caucasian (97%). The CTCL subtypes at diagnosis were MF in 104 (66.2%), pcALCL in 22 (14.0%), SS in 19 (12.1%) and other rare CTCL subtypes in 12 (7.6%).

Among 82 patients tested for CD30, 69 (84.1%) were found to express CD30 (Table 1).

CD30 values were <1% in 20.3%, 1–10% in 30.4%, >10% in 30.4% and unknown in 18.8%. In those patients with pcALCL as the primary subtype (data not shown), all 21 patients tested for CD30 were found to express CD30. For MF, SS or other rare CTCL subtypes, CD30 expression in those tested was 85.1%, 66.7% and 50%, respectively. In terms of T-cell receptor (TCR) gene rearrangement, 74 patients received a skin test and 54 a blood test. Among those who received the skin test, TCR gene rearrangement was clonal in 68 (91.9%) and polyclonal/oligoclonal in six (8.1%). Among those who received the blood test, TCR gene rearrangement was clonal in 33 (61.1%) and polyclonal/oligoclonal in 21 (38.9%). The TCR gene rearrangement in the skin was clonal in 90.2–100% across four primary subtypes (i.e., pcALCL, MF, other rare CTCL subtypes and SS). In contrast, TCR gene rearrangement in the blood was variable across primary subtypes (clonal: 28.6% in pcALCL, 51.9% in MF, 71.4% in other rare CTCL subtypes and 92.3% in SS).

### 3.1. Disease Stage across Lines of Therapy

The ISCLC/EORTC stage for MF and SS and TNMB stage for non-MF and non-SS subtypes are presented in Table 2. At diagnosis, the proportion of patients with advanced CTCL was 39.0% for MF and SS (stage IIB–IV) compared to 32.4% for non-MF and non-SS subtypes (T3-T4). A shift towards higher stages was observed (upstaging) with increasing lines of therapy. Indeed, ISCLC/EORTC stages I, II, III and IV changed from 44.7%, 18.7%, 8.1% and 15.4% at diagnosis, to 34.1%, 26.1%, 9.8% and 17.1% at first-line treatment initiation, respectively. The observed 10.6% decrease in stage I coincided with a 10.8% increase in stages II, III and IV. Upstaging continued at the time of second-line treatment initiation. Indeed, ISCLC/EORTC stage I, II, III and IV were 26.3%, 28.8%, 13.6% and 17.8%, respectively, indicating a 7.8% decrease in stage I and a coinciding increase of 7.2% in stages II–IV. At the time of second-line treatment initiation (i.e., first R/R population), the proportion of patients with advanced stages was also higher than at diagnosis (MF/SS: 55.9% vs. 39.0%; non-MF/non-SS: 36.4% vs. 32.4%, respectively). At the time of third-line treatment initiation, too many patients had been excluded from the study population (due to patients not being treated after second R/R or treatment being unknown) to be able to interpret the changes observed.

### 3.2. Treatments by Lines of Therapy

**Figure 1:**
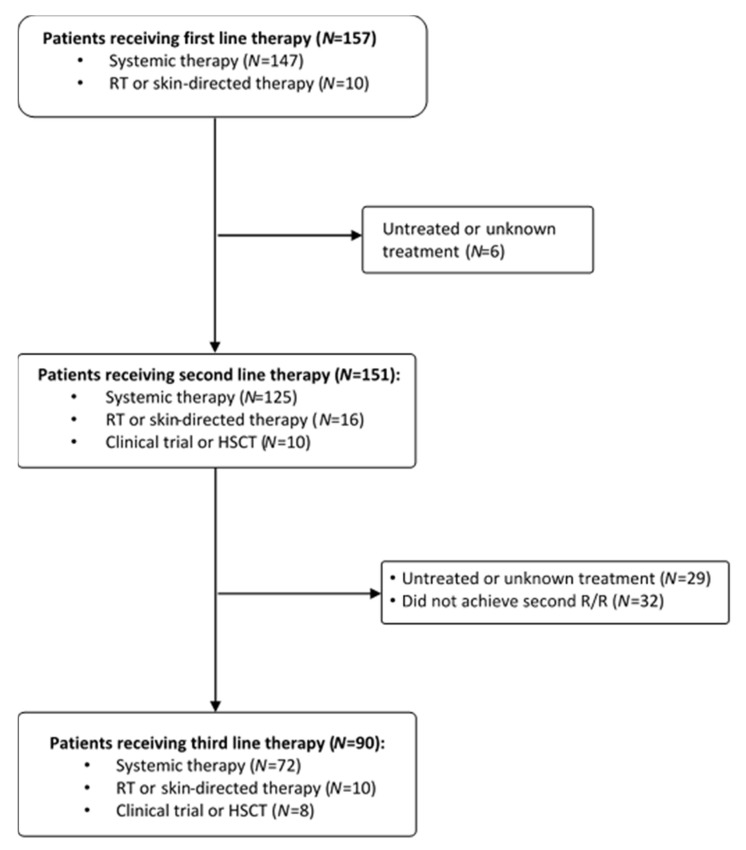
Patient flow diagram across lines of therapy. Abbreviations: HSCT: hematopoietic stem-cell transplantation; R/R: relapsed/refractory; RT: radiotherapy.

The patient flow diagram across the three lines of therapy is shown in Figure 1. Among the 157 included patients, 151 proceeded to second-line and 90 to third-line therapy. Six patients who were either untreated or whose treatment was unknown were excluded from the second-line population. Sixty-one patients who were untreated, had an unknown treatment or did not relapse or become refractory after second-line treatment were excluded from the third-line population. The number of patients who received systemic therapy within routine practice (with or without RT/skin-directed therapy) was 147 in the first line, 125 in the second line and 72 in the third line. The number of patients who received RT alone or local skin-directed alone was 10 in the first line, 16 in the second line and 10 in the third line. Finally, the number of patients who received a HSCT or were treated in a clinical trial was 10 in the second and 8 in the third line.

#### 3.2.1. First-Line Therapy

At the initiation of first-line therapy, 51/157 (32.5%) patients developed new lesions in addition to those present at diagnosis. At that time, the majority of the study population had skin symptoms (i.e., skin symptoms present in 115 (73.2%), absent in 31 (19.7%) and unknown in 11 (7.0%)). The three most common skin symptoms were pruritus/itching in 91 (58.0%), rash in 50 (31.8%) and irritation/burning in 61 (38.9%). The most serious skin symptoms included weeping lesions in two (1.3%), ulceration in three (1.9%), tumors in three (1.9%) and other types of skin cancer in two (1.3%).

A total of 147 patients (93.6%) received systemic therapy as first-line treatment, alone in 47/147 (32.0%), with RT in 12/147 (8.2%), with local skin-directed therapy in 67/147 (45.6%) or with a combination of RT and local skin-directed therapy in 21/147 (14.3%). A total of 10 patients received RT alone or RT with skin-directed therapy. Overall, systemic therapy with local skin-directed therapy was the most common treatment in the first line.

The details of the various types of systemic therapies, RT and skin-directed therapies received in the first line are presented in Table 3.

The regimen details of the systemic therapies (excluding ECP) received by 147 patients and used by two or more patients in the first line are presented in Table 4. Systemic therapy was diverse in the first line (Table 3), including chemotherapy (i.e., methotrexate, single conventional cytotoxic agent and combination of conventional cytotoxic agents) in 67 (45.6%), retinoids including bexarotene in 39 (26.5%), IFN in 31 (21.1%), ECP in 4 (2.7%), corticosteroids in 3 (2.0%) and novel agents in 3 (2.0%). Single-agent chemotherapy (excluding methotrexate) was administered to 23 (15.6%), either alone or in combination with bexarotene or prednisone (Table 3). Single agents received by more than one patient included gemcitabine in eight (5.4%), chlorambucil in six (4.1%) and pegylated liposomal doxorubicin in six (4.1%) (Table 4). Combination chemotherapy was received by 21 (14.3%) and was often administered along with prednisone, methylprednisolone, bexarotene or both a corticosteroid and bexarotene (Table 3). In the first line, combination chemotherapies were either CHOP/CHOP-like (12.2%) or EPOCH/EPOCH-like (2%). Finally, the new agents alemtuzumab, brentuximab vedotin and bortezomib were received by one patient each, either alone or with chemotherapy. Among the 89 patients who received skin-directed therapy, PUVA phototherapy was the most common non-topical therapy and was used in 43 (48.3%) patients (Table 3). Topical therapies included corticosteroids, nitrogen mustard (i.e., carmustine) and mechlorethamine. RT was most commonly electron beam, used in 22/43 (51.2%), and its extent was local in 37/43 (86.0%) and total skin (TSEBT) in 6/43 (14.0%).

##### Outcomes of First-Line Therapy: Clinical Response, Skin Symptoms and Time to First R/R

Following first-line therapy, 128 patients had a known global response, including a complete response in 22 (14.0%), a partial response in 46 (29.3%), a stable disease in 20 (12.7%) and progressive disease in 40 (25.5%) (Table 5). Among the 29 patients whose global response was unknown, 17 had a known skin response (i.e., three (1.9%) complete, eight (5.1%) partial, five (3.2%) stable and one (0.6%) progression). ORR based on global and skin response was 79/157 (50.3%). Overall, 41/157 (26.1%) experienced disease progression. These estimates are conservative due to 12 patients with an unknown response being included in the denominator.

After first-line treatment, skin symptoms were present in 101 (64.3%), absent in 35 (22.3%) and unknown in 21 (13.4%). The three most common skin symptoms were again pruritus/itching in 77 (49.0%), rash in 36 (22.9%) and irritation/burning in 56 (35.7%), but, notably, the prevalence was lower compared to when first-line treatment was initiated. The most serious skin symptoms observed included weeping lesions in two (1.3%), ulceration in two (1.3%), tumors in three (1.9%) and other types of skin cancer in three (1.9%).

All patients who received a first-line therapy eventually relapsed or became refractory, as per the inclusion criteria. The median time to first R/R was 10.3 months (range: 0.3–255.1).

#### 3.2.2. Second-Line Therapy

Among the 151 patients who initiated second-line therapy, 91 (60.3%) developed new lesions at the time of treatment initiation. The median age was 60.0 years (range: 19.0–98.0). A total of 125 patients (82.8%) received systemic therapy as second-line treatment, alone in 50/125 (40.0%), with RT (with or without skin-directed therapy) in 30/125 (24.0%) or with local skin-directed therapy in 45/125 (36.0%). A total of 16 patients received RT alone, skin-directed alone or RT with skin-directed therapy. Overall, systemic therapy alone was the most common therapy in the second line.

The various types of therapies received in the second line are presented in Table 3. The details of the systemic therapies received by 125 patients in the second line are presented in Table 4. Systemic therapies included chemotherapy in 59 (47.2%), retinoids (including bexarotene) in 33 in (26.4%), IFN in 14 (11.2%), novel agents in 10 (8.0%), ECP in 5 (4.0%), corticosteroids in 2 (1.6%) and HDAC-inhibitors in 2 (1.6%) (Table 3). An uptake in the use of novel agents was observed in the second line. Novel agents consisted of alemtuzumab in two (1.6%) and brentuximab vedotin in eight (6.4%). Single-agent chemotherapies were similar to those encountered in the first line, with a predominance of gemcitabine, which was used in 18 patients (Table 4). As in the first line, these single agents were often used along with prednisone, acitretin, IFN, methotrexate and bexarotene. In contrast to the first line, there was a multitude of different combination chemotherapies in the second line. Combination chemotherapies included CHOP in 4.8% of patients. The remainder received a mosaic of combination chemotherapies, including cyclophosphamide-based combinations, gemcitabine-based regimens, cisplatin–cytarabine, thiotepa-busulfan and bendamustine-pegylated liposomal doxorubicin. These regimens were again often used with bexarotene, IFN, methotrexate, prednisone, dexamethasone or methylprednisolone. The use of brentuximab vedotin was more prevalent in the second line compared to the first line (8 versus 1, respectively), most often with another chemotherapy agent. PUVA phototherapy was the most common therapy besides topical therapies. Similar to observations in the first line, topical treatment consisted of steroids, carmustine and mechlorethamine. RT was most commonly electron beam, as opposed to X-ray seen in the first line. RT was local in 72.7% and total in 20.5%.

##### Outcomes of Second-Line Therapy: Clinical Response, Skin Symptoms, Toxicity and Time to R/R

Following second-line therapy, clinical response was evaluated for 141 patients who received systemic therapy (125), RT (14) or local skin-directed therapy (2). Global response was known in 108 patients (Table 5) and included a complete response in 20 (14.2%), a partial response in 41 (29.1%), stable disease in 18 (12.8%) and progressive disease in 29 (20.6%). In the 33 patients whose global response was unknown, local skin response was known in 18. ORR based on global and skin response was 51.1%. Overall, at least 23.4% of patients experienced disease progression based on known responses recorded. Based on data from 119 patients, the median time to second R/R was 11.2 months (range: 0–174.6). In those patients (*n* = 61) who received bexarotene or methotrexate (with or without other therapies), ORR based on global and skin response was 47.5%. As many as 31.2% of these patients experienced progression.

Among the 141 patients, skin symptoms were present in 67 (47.5%) and absent in 52 (36.9%). The three most common symptoms were again pruritus/itching in 52 (36.9%), irritation/burning in 33 (23.4%) and rash in 31 (22.0%). No weeping lesions were observed after second-line therapy. Ulceration, tumors and other types of skin cancers were observed in one patient each.

Among the 141 patients who received systemic therapy, RT or skin-directed therapy in the second line, non-skin-related adverse events were reported to be present in 45 (31.9%) and absent in 71 (50.4%). Hematologic adverse events included febrile neutropenia (14.9%), anemia (7.8%), leukopenia (3.5%), neutropenia (2.8%) and thrombocytopenia (2.8%). The most common non-hematologic adverse events (present in >10 patients) included alopecia (18.4%), fatigue/asthenia (12.1%) and anorexia (7.1%). Serious adverse events included sepsis and pulmonary embolism, which were experienced by one patient each.

#### 3.2.3. Third-Line Therapy

Among the 90 patients who initiated third-line therapy, 53 (58.9%) developed new lesions, indicating a worsening of disease in the majority of patients. A total of 72 patients received systemic therapy in the third line, alone in 34/72 (47.2%), with RT (with or without skin-directed therapy) in 16/72 (22.2%) or with skin-directed therapy alone in 22/72 (30.6%). A total of 10 patients received RT alone, RT with skin-directed therapy or skin-directed therapy alone.

The various types of therapies in the third line are summarized in Table 3. Systemic therapy agents received by 72 patients in the third line are presented in Table 4. Systemic therapies included chemotherapy in 31 (43.1%), retinoids (including bexarotene) in 13 (18.1%), IFN in 11 (15.3%) and a novel agent in 11 (15.3%) (Table 3). As in the second line, there was a multitude of combination chemotherapies, including CHOP, EPOCH, cyclophosphamide-based regimens, gemcitabine-based regimens and vincristine–vinblastine–mitoxantrone. Dexamethasone, bexarotene, prednisone and methylprednisolone were often administered with combination chemotherapy. Again, as in previous lines, PUVA was the most common skin-directed therapy besides topical therapies. RT was local in 68.0% and total in 32.0% of patients.

##### Outcomes of Third-Line Therapy: Clinical Response, Skin Symptoms, Toxicity and Time to R/R

Following third-line therapy, clinical response was evaluated in 82 patients who received systemic therapy (72), RT (9) or local skin-directed therapy (1). Global response was known in 60 patients (Table 5) and included a complete response in 12 (14.6%), a partial response in 20 (24.4%), stable disease in 11 (13.4%) and progressive disease in 17 (20.7%). In the 22 patients whose global response was unknown, local skin response was known in 11. ORR based on global and skin response was 45.1%. Overall, at least 20 (24.4%) experienced progressive disease. Based on 59 patients, the median time to a third R/R was 7.9 months (range 0.0–39.0).

Among the 82 patients, skin symptoms were present in 33 (40.2%), pruritus/itching in 19 (23.2%), irritation/burning in 15 (18.3%) and rash in 11 (13.4%), being the three most common skin symptoms.

Among the 82 patients who received systemic therapy, RT or skin-directed therapy in the third line, non-skin-related adverse events were present in 18 (22.0%) and absent in 48 (58.5%). The most common hematologic adverse events included febrile neutropenia (12.2%), anemia (6.1%) and leukopenia (4.9%). The most common non-hematologic adverse event (present in > 10 patients) was alopecia (15.9%). Serious adverse events included sepsis in two (2.4%) and both pulmonary embolism and renal failure in one patient.

### 3.3. Treatment Pattern Focusing on Systemic Therapies

The broad treatment pattern (including no treatment) across the three lines of therapy is summarized in Table 6. The number of patients treated with chemotherapy, retinoids and IFN decreased with increasing lines of therapy, while the number of untreated patients increased with increasing lines of therapy. The number of patient treated with novel agents and HDAC inhibitors trended towards a slight increase with increasing lines of therapy.

### 3.4. Survival Outcomes

**Figure 2:**
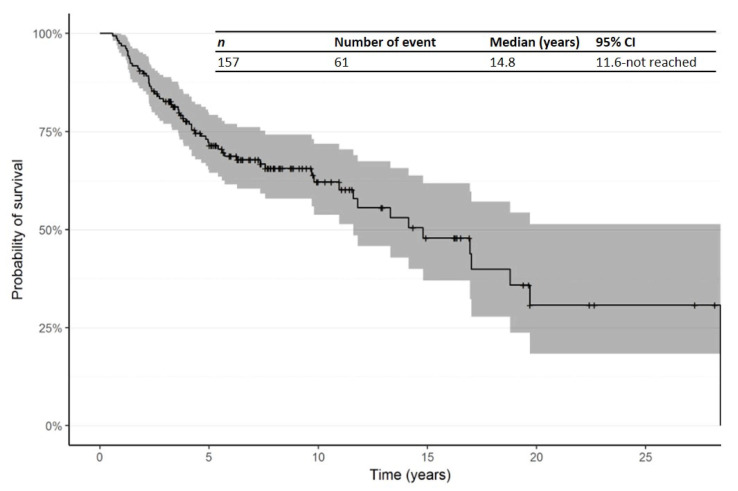
Kaplan–Meier curve for overall survival (OS) from time of diagnosis. Abbreviations: CI: confidence interval; OS: overall survival.

At the date of last follow-up, 61 (38.9%) had died, 93 (59.2%) were alive and 3 (1.9%) had unknown survival status. The median age at time of final status was 67.0 years (range: 25.0–85.0). The median duration of follow-up since time of first R/R was 3.2 years (Range: 0.0–26.0 years; IQR: 1.8–5.5 years). The main causes of death were CTCL (52.5%), CTCL complications or toxicity (18.0%), other causes (19.7%) and unknown (9.8%). The Kaplan–Meier curve for OS from time of diagnosis is shown in Figure 2. The median OS from time of diagnosis was 14.8 years (95% CI: 11.6—not reached).

**Figure 3:**
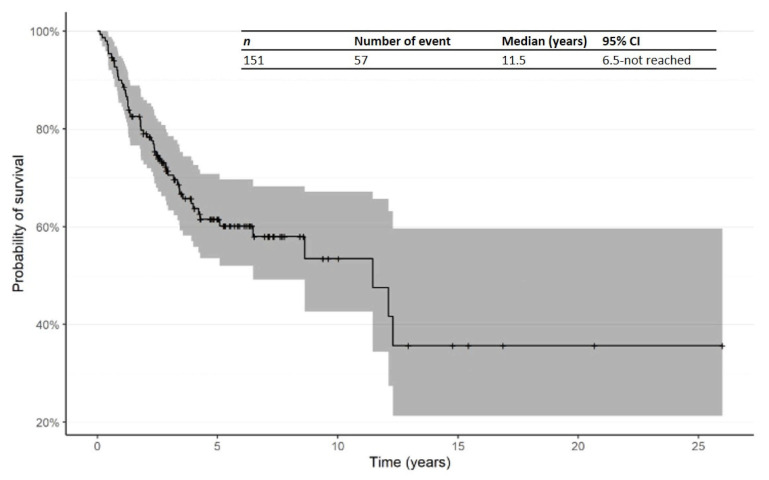
Kaplan–Meier curve for OS from time of first relapsed/refractory in patients receiving second-line therapy. Abbreviations: CI: confidence interval; OS: overall survival; R/R: relapsed/refractory.

**Figure 4:**
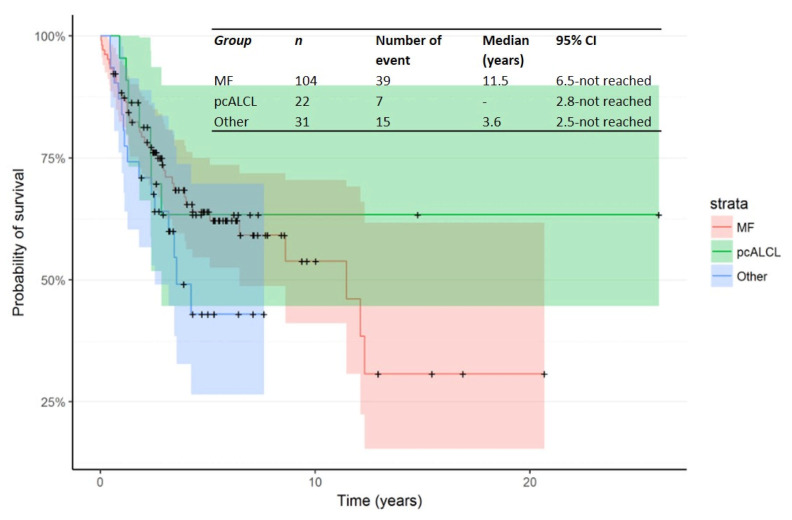
Kaplan–Meier curve for OS from time of R/R1 by primary subtype.

The Kaplan–Meier curve for OS from time of first R/R in patients treated in the second line is shown in Figure 3. The median OS from time of first R/R was 11.5 years (95% CI: 6.5—not reached). In addition, Kaplan–Meier curves for OS from time of R/R1 by primary subtype for Mf, pcALCL and others are shown in Figure 4.

**Figure 5:**
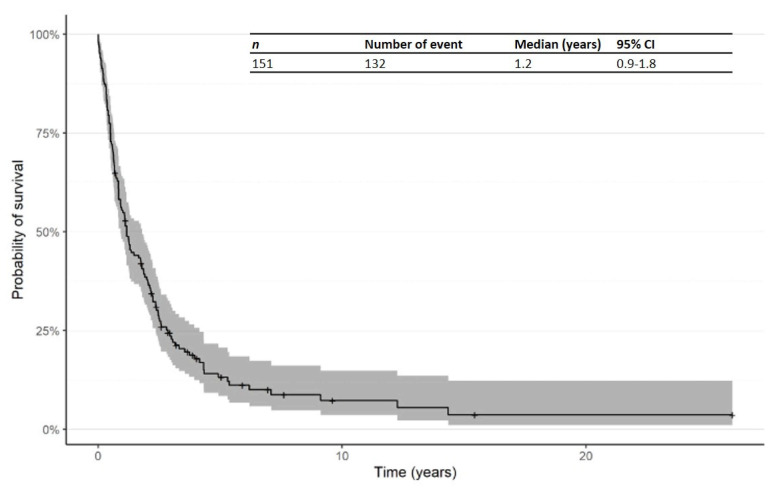
Kaplan–Meier curve for progression-free survival (PFS) from time of first R/R in patients receiving second-line therapy. Abbreviations: CI: confidence interval; PFS: progression-free survival; R/R: relapsed/refractory.

The Kaplan–Meier curve for PFS from time of first R/R in patients treated in the second line is shown in Figure 5. The median PFS from time of first R/R was 1.2 years (95% CI: 0.9–1.8).

## 4. Discussion

Despite interest in the treatment of R/R CTCL, recent studies have mostly focused on the treatment of advanced MF and SS and less on the treatment of R/R. There is also a paucity of data on the treatment and outcomes of pcALCL and other rare CTCL subtypes. This retrospective study aimed to enhance our understanding of treatments and outcomes in R/R CTCL, including pcALCL and other rare subtypes. There was a significant level of heterogeneity in treatment types, which included mainly chemotherapies (including methotrexate), retinoids, IFN, PUVA phototherapy, RT and topical therapies. In this cohort of R/R CTCL patients, chemotherapy was the most common systemic therapy in second- and third-line therapy (47.2% and 43.1%, respectively). There was a wide variety of different chemotherapy approaches in the second and third line, in sharp contrast to the fewer, more established chemotherapy approaches in the first line (i.e., gemcitabine, chlorambucil, pegylated doxorubicin, CHOP/CHOP-like and EPOCH/EPOCH-like). These findings highlight the lack of consensus in the first line, which is magnified further beyond the first-line treatment, as well as the urgent unmet treatment needs in second- and third-line approaches. A modest uptake was observed in the use of novel agents and HDAC inhibitors in the second and third line compared to the first line, indicating that these agents are possibly starting to be used in some parts of the world. In contrast to a large international retrospective study that found that ECP, bexarotene, phototherapy and methotrexate were the top four first-line treatments, in our study, chemotherapy (single agents and CHOP/CHOP-like), PUVA phototherapy, retinoids and IFN were the four most common first-line treatments [19]. This highlights the importance of chemotherapy in the treatment of advanced CTCL in Europe compared to the US and other parts of the world. Overall, there was clear evidence of disease progression at each new line of therapy (e.g., upstaging and appearance of new lesions), but treatments were ineffective in halting progression for many patients. Skin symptoms persisted across the different lines of therapy. The increased use of chemotherapy, however, was associated with significant hematological toxicity (e.g., neutropenia, febrile neutropenia, anemia and thrombocytopenia) as well as non-hematological adverse events (e.g., infection, anorexia, diarrhea, vomiting and nausea).

Considering the heterogeneity of treatments and CTCL subtypes, ORR was remarkably similar at 45–50% across all three lines of therapy. This is within the range of ORR estimates reported in clinical trials and observational studies. Indeed, in the literature, estimates of ORR for the relevant therapies were reported to be: 39–66% for bexarotene, 31–68% for gemcitabine, 41–83% for pegylated liposomal doxorubicin, 33–58% for methotrexate and 29–66% for IFN [1,20]. Overall, the ORR estimates found in this study are consistent with these estimates.

Disease progression was found to be approximately 25%, indicating that, despite treatment, a quarter of R/R patients eventually relapse or become refractory. Furthermore, the median time to subsequent relapse was less than one year across all three lines, i.e., 10.3 months (range: 0–255) in the first line, 11.2 months (range: 0–175) in the second line and 7.9 months (range: 0–39) in the third line. In a retrospective study of 198 patients diagnosed with MF/SS and requiring systemic therapy, Hughes et al., 2015 estimated that the median time to next treatment (TTNT) was 5.4 months (95%CI: 5.1–6.1) for the overall study population [21]. Time to R/R and TTNT are different and thus comparisons are difficult. However, our estimate of 10.3 months could be higher for a few possible reasons. First, the treatment types differed in our study and one key difference was the much higher use of bexarotene in our study compared to the study of Hughes et al., 2015 [21]. According to Hughes et al., 2015, TTNT for bexarotene was higher at 7.3 months (95% CI: 2.6–110.8) compared to the median TTNT for all study treatments combined (i.e., 5.4 months). These results could explain the findings of a longer time to R/R in our study. Perhaps more importantly, TTNT is known to be affected by the physician’s tendency to treat patients regardless of response to treatment. Therefore, it is perhaps not surprising that TTNT and time to R/R are somewhat different.

We acknowledge that there were some limitations to this study due to the retrospective nature of the study. There were missing response data, such that ORR estimates are likely to be conservative and underestimate actual responses. In addition, response criteria were based on investigators’ assessment using data available in the medical chart.

This observational study provided real-world data on the current treatments used in the management of CTCL across three lines of therapy. Combination therapies were more often used in clinical practice, in line with EORTC-CTCL guidelines [14]. Despite the wide application of chemotherapy to 45% of patients, skin symptoms remained common, a limited number of complete responses were achieved, and patients experienced progression or death after treatment in each of the three lines. Our findings indicate that the clinical burden of CTCL is likely to be considerable in Europe, and, in this context, recently approved targeted agents may assist in addressing this problem. Use of targeted agents was uncommon and treatment patterns might change as these agents become more widely adopted in routine practice. Further research is warranted to assess how clinical practice and outcomes will evolve with recently approved targeted therapies such as brentuximab vedotin and mogamulizumab.

## 5. Conclusions

This European observational study provided real-world data on the current treatments used in the management of CTCL across three lines of therapy. As a result, there was a significant level of heterogeneity in treatment types than expected by European guidelines. These findings highlight the lack of consensus in relapsed/refractory CTCL, as well as the lack of consensus already in the R/R first line, which is magnified further beyond the first-line treatment, as well as the urgent unmet treatment needs in second- and third-line approaches.

## Figures and Tables

**Table 1:** Patient demographics and clinical characteristics at diagnosis (*n* = 157).

Variable	*n* (%)
Age at diagnosis	-
Mean (SD)	55.7 (15.4)
Median (range)	56.0 (19.0–97.0)
Gender, *n* (%)	-
Male	99 (63.1)
Female	58 (36.9)
Ethnicity *, *n* (%)	-
Caucasian	120 (76.4)
African	1 (0.6)
Caribbean	1 (0.6)
Other	2 (1.3)
Unknown	33 (21.0)
Primary subtype at diagnosis, *n* (%)	-
MF	104 (66.2)
pcALCL	22 (14.0)
SS	19 (12.1)
Other CTCL subtypes:	31 (19.7)
Primary cutaneous peripheral T-cell lymphoma, unspecified	6 (3.8)
Primary cutaneous CD4+ small/medium T-cell lymphoma	2 (1.3)
Lymphomatoid papulosis	1 (0.6)
Other rare subtypes	3 (1.9)
CD30 tested, yes ^†^	82 (52.2)
CD30 expressed	69 (84.1)
CD30 < 1%	14 (20.3)
CD30 1–10%	21 (30.4)
CD30 > 10%	21 (30.4)
CD30 unknown	13 (18.8)
TCR gene rearrangement skin tested, yes ^‡^	74 (47.1)
Clonal	68 (91.9)
Polyclonal/oligoclonal	6 (8.1)
TCR gene rearrangement blood tested, yes ^≠^	54 (34.4)
Clonal	33 (61.1)
Polyclonal/oligoclonal	21 (38.9)

Abbreviations: MF: mycosis fungoides; pcALCL: primary cutaneous anaplastic large-cell lymphoma; SS: Sézary syndrome; SD: standard deviations; TCR: T-cell receptor; * Ethnicity data were not collected in France; ^†^ CD30 not tested: 52 (33.1%); CD30 testing unknown: 23 (14.6%); ^‡^ TCR gene rearrangement skin not tested: 50 (31.8%); TCR gene rearrangement skin testing unknown: 33 (21.0%); ^≠^ TCR gene rearrangement blood not tested: 63 (40.1%); TCR gene rearrangement blood testing unknown: 40 (25.5%).

**Table 2:** Disease stage at diagnosis and prior to treatment initiation in 1st, 2nd and 3rd line.

Variable	Diagnosis	1st Line	2nd Line	3rd Line
	*n* (%)	*n* (%)	*n* (%)	*n* (%)
**ISCL/EORTC staging, MF and SS;** **N**	**123**	**123**	**118**	**70**
IA	12 (9.8)	8 (6.5)	5 (4.2)	2 (2.9)
IB	43 (35.0)	34 (27.6)	26 (22.0)	19 (27.1)
IIA	4 (3.3)	5 (4.1)	5 (4.2)	2 (2.9)
IIB	19 (15.4)	27 (22.0)	29 (24.6)	19 (27.1)
IIIA	2 (1.6)	5 (4.1)	11 (9.3)	3 (4.3)
IIIB	8 (6.5)	7 (5.7)	5 (4.2)	2 (2.9)
IVA	17 (13.8)	20 (16.3)	19 (16.1)	15 (21.4)
IVB	2 (1.6)	1 (0.8)	2 (1.7)	2 (2.9)
Unknown	16 (13.0)	16 (13.0)	16 (13.6)	5 (7.1)
**TNM staging, pcALCL and other non-MF/non-SS; N**	**34**	**34**	**33**	**20**
**T (skin)**	-	-	-	-
T0	1 (2.9)	1 (2.9)	2 (6.1)	2 (10.0)
T1	10 (29.4)	10 (29.4)	7 (21.2)	2 (10.0)
T2	6 (17.6)	6 (17.6)	5 (15.2)	5 (25.0)
T3	9 (26.5)	9 (26.5)	11 (33.3)	6 (30.0)
T4	2 (5.9)	2 (5.9)	1 (3.0)	2 (10.0)
Unknown	6 (17.6)	6 (17.6)	7 (21.2)	3 (15.0)
**N (lymph node)**	-	-	-	-
N0	21 (61.8)	20 (58.8)	12 (36.4)	9 (45.0)
N1	2 (5.9)	3 (8.8)	3 (9.1)	0 (0.0)
N2	3 (8.8)	3 (8.8)	6 (18.2)	4 (20.0)
N3	2 (5.9)	2 (5.9)	5 (15.2)	3 (15.0)
Nx	0 (0.0)	0 (0.0)	0 (0.0)	0 (0.0)
Unknown	6 (17.6)	6 (17.6)	7 (21.2)	4 (20.0)
**M (viscera)**	-	-	-	-
M0	26 (76.5)	26 (76.5)	24 (72.7)	15 (75.0)
M1	2 (5.9)	2 (5.9)	3 (9.1)	0 (0.0)
**Unknown**	6 (17.6)	6 (17.6)	6 (18.2)	5 (25.0)

Abbreviations: ISCL: International Society for Cutaneous Lymphomas; EORTC: European Organisation for Research and Treatment of Cancer; MF: mycosis fungoides; pcALCL: primary cutaneous anaplastic large-cell lymphoma; SS: Sézary syndrome. Bolded variables signify subheadings.

**Table 3:** Systemic therapies, radiotherapy (RT) and skin-directed therapies across 3 lines of therapy.

Systemic Treatment	1st Line (*n* = 157)		2nd Line (*n* = 151)		3rd Line (*n* = 90)	
	*n*	%	*n*	%	*n*	%
**Systemic therapy, *N***	**147**	-	**125**		**72**	-
Bexarotene	25	17.0	27	21.6	9	12.5
Methotrexate	23	15.6	18	14.4	7	9.7
Other retinoids	14	9.5	6	4.8	4	5.6
Single-agent chemotherapy ^†^	23	15.6	25	20.0	15	20.8
Combination chemotherapy	21	14.3	16	12.8	9	12.5
IFN	31	21.1	14	11.2	11	15.3
Corticosteroid	3	2.0	2	1.6	0	0.0
HDAC inhibitors	0	0.0	2 ^‡^	1.6	2 ^§^	2.8
ECP	4	2.7	5	4.0	4	5.6
Novel agents	3	2.0	10	8.0	11	15.3
**Radiotherapy, *N***	**43**	-	**44**	-	**25**	-
Electron beam	22	51.2	20	45.5	14	56.0
X-ray	16	37.2	18	40.9	11	44.0
Unknown	5	11.6	6	13.6	0	0.0
Skin-directed therapy *, *N*	**89**	-	**68**	-	**28**	-
Topical steroids and other	63	70.8	53	77.9	27	96.4
Imiquimod	1	1.1	0	0.0	0	0.0
Photodynamic	0	0.0	1	1.5	1	3.6
PUVA phototherapy	43	48.3	24	35.3	5	17.9
UVB phototherapy	2	2.2	1	1.5	1	3.6
nbUVB phototherapy	7	7.9	5	7.4	1	3.6
Other	7	7.9	3	4.4	2	7.1

Abbreviations: ECP: extracorporal photopheresis; HDAC: histone deacetylase; IFN: interferon; nb: narrow band; PUVA: psoralen and ultraviolet A; RT: radiotherapy; UVB: ultra-violet B. ^†^ All mono-chemotherapy other than methotrexate; * multiple selections were possible; ^‡^ 1 patient in Spain and 1 in the UK; ^§^ 1 patient in France and 1 in Italy. Notes: The bexarotene group was defined as bexarotene alone or with IFN, another retinoid, a corticosteroid or methotrexate. The methotrexate group was defined as methotrexate alone or with IFN or a corticosteroid. The other retinoids included acitretin, isotretinoin (rarely) or all-trans retinoic acid (rarely) or the same agents with IFN, methotrexate or a corticosteroid. Single-agent chemotherapy included bendamustine, chlorambucil, cyclophosphamide, gemcitabine, etoposide, vinblastine, pegylated liposomal doxorubicin/doxorubicin or any of these agents with bexarotene, prednisone, IFN or methotrexate. Combination chemotherapy included single chemotherapy agents with or without bexarotene, dexamethasone, methylprednisolone, prednisone, IFN or methotrexate. The IFN group included IFN-α alone, IFN-γ alone or IFN with a corticosteroid. The HDAC inhibitors included resminostat, vorinostat and romidepsin (1 patient each). The ECP group included ECP alone or in combination with any other therapy (including chemotherapy). Novel agents included alemtuzumab, brentuximab vedotin and bortezomib groups alone or in combination with any other therapy (including chemotherapy). Bolded variables signify subheadings.

**Table 4:** Regimens used by 2 or more patients in at least one line of therapy.

Systemic Treatment	1st Line (*n* = 157)		2nd Line (*n* = 151)		3rd Line (*n* = 90)	
	*n*	%	*n*	%	*n*	%
Systemic therapy, *N*	147	-	125	-	72	-
Bexarotene	25	17.0	27	21.6	9	12.5
Methotrexate	23	15.6	18	14.4	7	9.7
Acitretin	13	8.8	6	4.8	3	4.2
Gemcitabine	8	5.4	18	14.4	5	6.9
Chlorambucil	6	4.1	3	2.4	1	1.4
Pegylated liposomal doxorubicin	6	4.1	3	2.4	2	2.8
Doxorubicin	0	0.0	1	0.8	2	2.8
CHOP/CHOP-like	18	12.2	6	4.8	3	4.2
EPOCH/EPOCH-like	3	2.0	0	0.0	1	1.4
IFN-α	29	19.7	14	11.2	11	15.3
IFN-γ	2	1.4	0	0.0	0	0.0
Alemtuzumab	1	0.7	2	1.6	3	4.2
Brentuximab vedotin	1	0.7	8	6.4	7	9.7
Bortezomib	1	0.7	0	0.0	1	1.4
Total number used in ≥2 patients	136	92.5	106	84.8	55	76.4

Abbreviations: CHOP: cyclophosphamide, doxorubicin, vincristine and prednisolone; ECP: extracorporal photopheresis; EPOCH: etoposide, vincristine, doxorubicin, cyclophosphamide and prednisolone; IFN: interferon.

**Table 5:** Response to systemic therapy in 1st, 2nd and 3rd line.

Clinical Response	1st Line (*n* = 157)	2nd Line (*n* = 151)	3rd Line (*n* = 90)
	*n* (%)	*n* (%)	*n* (%)
Response assessment population, N	157	141 *	82 ^†^
Global response (known), N	128	108	60
Complete	22 (14.0)	20 (14.2)	12 (14.6)
Partial	46 (29.3)	41 (29.1)	20 (24.4)
Stable	20 (12.7)	18 (12.8)	11 (13.4)
Progression	40 (25.5)	29 (20.6)	17 (20.7)
Unknown global response	29 (18.5)	33 (23.4)	22 (26.8)
Local skin response (known), N	17	18	11
Complete	3 (1.9)	2 (1.4)	0 (0.0)
Partial	8 (5.1)	9 (6.4)	5 (6.1)
Stable	5 (3.2)	3 (2.1)	3 (3.7)
Progression	1 (0.6)	4 (2.8)	3 (3.7)
Unknown skin response	12 (7.6)	15 (10.6)	11 (13.4)
**ORR**	**79 (50.3)**	**72 (51.1)**	**37 (45.1)**
**Progression**	**41 (26.1)**	**33 (23.4)**	**20 (24.4)**

Abbreviations: ORR: overall response rate. * All patients who received systemic therapy (125), radiotherapy (14) or skin-directed therapy (2) within routine practice were assessed for response to 2nd-line therapy. The response of patients who were treated within a clinical trial or received an HSCT in 2nd line was not assessed. ^†^ All patients who received systemic therapy (72), radiotherapy (9) or skin-directed therapy (1) within routine practice were assessed for response to 3rd-line therapy. The response of patients who were treated within a clinical trial or received an HSCT in 3rd line was not assessed. Bolded variables signify subheadings.

**Table 6:** Treatment pattern across the 3 lines of therapy.

Treatment Type	1st Line		2nd Line		3rd Line	
	*n*	%	*n*	%	*n*	%
Total, *N*	157	100	157	100	157	100
Chemotherapy ^†^	67	42.7	59	37.6	31	19.7
Retinoids ^‡^	39	24.8	33	21.0	13	8.3
IFN and corticosteroids	34	21.7	16	10.2	11	7.0
ECP	4	2.5	5	3.2	4	2.5
HDAC inhibitors or novel agents	3	1.9	12	7.6	13	8.3
RT alone and/or skin-directed therapy alone	10	6.4	16	10.2	10	6.4
HSCT or clinical trial *	0	0.0	10	6.4	8	5.1
No treatment	0	0.0	6	3.8	67	42.7

Abbreviations: ECP: extracorporal photopheresis; HDAC: histone deacetylase; HSCT: hematopoietic stem cell transplant; IFN: interferon; RT: radiotherapy. * As per the eligibility criteria, HSCT and treatment within a clinical trial were not allowed in 1st line. ^†^ Chemotherapy includes conventional single-agent and combination chemotherapy as well as methotrexate. ^‡^ Retinoids include bexarotene, acitretin, isotretinoin and all-trans retinoic acid.

## Data Availability

The data presented in this study are available on request from the corresponding author.

## References

[1] Zinzani P.L., Bonthapally V., Huebner D., Lutes R., Chi A., Pileri S. (2016). Panoptic clinical review of the current and future treatment of relapsed/refractory T-cell lymphomas: Cutaneous T-cell lymphomas. Crit. Rev. Oncol. Hematol..

[2] Olsen E.A. (2015). Evaluation, Diagnosis, and Staging of Cutaneous Lymphoma. Dermatol. Clin..

[3] Photiou L., van der Weyden C., McCormack C., Miles Prince H. (2018). Systemic Treatment Options for Advanced-Stage Mycosis Fungoides and Sézary Syndrome. Curr. Oncol. Rep..

[4] Willemze R., Jaffe E.S., Burg G., Cerroni L., Berti E., Swerdlow S.H., Ralfkiaer E., Chimenti S., Diaz-Perez J.L., Duncan L.M. (2005). WHO-EORTC classification for cutaneous lymphomas. Blood.

[5] European Federation of Pharmaceutical Industries and Associations (2016). Mycosis Fungoides. http://www.efpia.eu/diseases/15/59/Mycosis-Fungoides.

[6] Dobos G., Pohrt A., Ram-Wolff C., Lebbé C., Bouaziz J.D., Battistella M., Bagot M., de Masson A. (2020). Epidemiology of Cutaneous T-Cell Lymphomas: A Systematic Review and Meta-Analysis of 16,953 Patients. Cancers.

[7] Bradford P.T., Devesa S.S., Anderson W.F., Toro J.R. (2009). Cutaneous lymphoma incidence patterns in the United States: A population-based study of 3884 cases. Blood.

[8] Trautinger F., Eder J., Assaf C., Bagot M., Cozzio A., Dummer R., Gniadecki R., Klemke C.D., Ortiz-Romero P.L., Papadavid E. (2017). European Organisation for Research and Treatment of Cancer consensus recommendations for the treatment of mycosis fungoides/Sezary syndrome—Update 2017. Eur. J. Cancer.

[9] National Comprehensive Cancer Network (NCCN) Primary Cutaneous Lymphomas. https://www.nccn.org/guidelines/guidelines-detail?category=1&id=1491.

[10] Willemze R., Hodak E., Zinzani P.L., Specht L., Ladetto M., ESMO Guidelines Working Group (2013). Primary cutaneous lymphomas: ESMO Clinical Practice Guidelines for diagnosis, treatment and follow-up. Ann. Oncol..

[11] Schlaak M., Pickenhain J., Theurich S., Skoetz N., von Bergwelt-Baildon M., Kurschat P. (2012). Allogeneic stem cell transplantation versus conventional therapy for advanced primary cutaneous T-cell lymphoma. Cochrane Database Syst Rev..

[12] Lansigan F., Foss F.M. (2010). Current and emerging treatment strategies for cutaneous T-cell lymphoma. Drugs.

[13] Lymphoma Research Foundation (2014). Cutaneous T-Cell Lymphoma (CTCL). http://www.lymphoma.org/site/pp.asp?c=bkLTKaOQLmK8E&b=6300151.

[14] Willemze R., Hodak E., Zinzani P.L., Specht L., Ladetto M., on behalf of the ESMO Guidelines Committee (2018). Primary cutaneous lymphomas: ESMO Clinical Practice Guidelines for diagnosis, treatment and follow-up. Ann. Oncol..

[15] Prince H.M., Kim Y.H., Horwitz S.M., Dummer R., Scarisbrick J., Quaglino P., Zinzani P.L., Wolter P., Sanches J.A., Ortiz-Romero P.L. (2017). Brentuximab vedotin or physician’s choice in CD30-positive cutaneous T-cell lymphoma (ALCANZA): An international, open-label, randomised, phase 3, multicentre trial. Lancet.

[16] Bagot M. (2017). New Targeted Treatments for Cutaneous T-cell Lymphomas. Indian J. Dermatol..

[17] Kim Y.H., Bagot M., Pinter-Brown L., Rook A.H., Porcu P., Horwitz S.M., Whittaker S., Tokura Y., Vermeer M., Zinzani P.L. (2018). Mogamulizumab versus vorinostat in previously treated cutaneous T-cell lymphoma (MAVORIC): An international, open-label, randomised, controlled phase 3 trial. Lancet Oncol..

[18] Olsen E., Vonderheid E., Pimpinelli N., Willemze R., Kim Y., Knobler R., Zackheim H., Duvic M., Estrach T., Lamberg S. (2007). Revisions to the staging and classification of mycosis fungoides and Sezary syndrome: A proposal of the International Society for Cutaneous Lymphomas (ISCL) and the cutaneous lymphoma task force of the European Organization of Research and Treatment of Cancer (EORTC). Blood.

[19] Quaglino P., Maule M., Prince H.M., Porcu P., Horwitz S., Duvic M., Talpur R., Vermeer M., Bagot M., Guitart J. (2017). Global patterns of care in advanced stage mycosis fungoides/Sezary syndrome: A multicenter retrospective follow-up study from the Cutaneous Lymphoma International Consortium. Ann. Oncol..

[20] Alpdogan O., Kartan S., Johnson W., Sokol K., Porcu P. (2019). Systemic therapy of cutaneous T-cell lymphoma (CTCL). Chin. Clin. Oncol..

[21] Hughes C.F., Khot A., McCormack C., Lade S., Westerman D.A., Twigger R., Buelens O., Newland K., Tam C., Dickinson M. (2015). Lack of durable disease control with chemotherapy for mycosis fungoides and Sezary syndrome: A comparative study of systemic therapy. Blood.

